# Analysis of Anthrax Immune Globulin Intravenous with Antimicrobial Treatment in Injection Drug Users, Scotland, 2009–2010

**DOI:** 10.3201/eid2301.160608

**Published:** 2017-01

**Authors:** Xizhong Cui, Leisha D. Nolen, Junfeng Sun, Malcolm Booth, Lindsay Donaldson, Conrad P. Quinn, Anne E. Boyer, Katherine Hendricks, Sean Shadomy, Pieter Bothma, Owen Judd, Paul McConnell, William A. Bower, Peter Q. Eichacker

**Affiliations:** National Institutes of Health, Bethesda, Maryland, USA (X. Cui, J. Sun, P.Q. Eichacker);; Centers for Disease Control and Prevention, Atlanta, Georgia, USA (L.D. Nolen, C.P. Quinn, A.E. Boyer, K. Hendricks, S. Shadomy, W.A. Bower);; Glasgow Royal Infirmary, Glasgow, UK (M. Booth, L. Donaldson);; James Paget University Hospital, Norfolk, UK (P. Bothma);; Royal Derby Hospital, Derby, UK (O. Judd);; Crosshouse Hospital, Kilmarnock, UK (P. McConnell)

**Keywords:** Bacillus anthracis, anthrax, infection, treatment, anthrax immune globulin intravenous, AIG-IV, injection drug users, Scotland, bacteria, United Kingdom

## Abstract

We studied anthrax immune globulin intravenous (AIG-IV) use from a 2009–2010 outbreak of *Bacillus anthracis* soft tissue infection in injection drug users in Scotland, UK, and we compared findings from 15 AIG-IV recipients with findings from 28 nonrecipients. Death rates did not differ significantly between recipients and nonrecipients (33% vs. 21%). However, whereas only 8 (27%) of 30 patients at low risk for death (admission sequential organ failure assessment score of 0–5) received AIG-IV, 7 (54%) of the 13 patients at high risk for death (sequential organ failure assessment score of 6–11) received treatment. AIG-IV recipients had surgery more often and, among survivors, had longer hospital stays than did nonrecipients. AIG-IV recipients were sicker than nonrecipients. This difference and the small number of higher risk patients confound assessment of AIG-IV effectiveness in this outbreak.

## Abstract 2

Differences between recipients and nonrecipients and the small number of higher risk patients confounded assessment.

*Bacillus anthracis* is identified as a select agent subject to select agent regulations (*1*–*3*) and as a potential bioweapon that presents a high risk to the US public (*4*,*5*). Production of lethal toxins and edema toxins by *B. anthracis* is central to the bacterium’s pathogenesis (*6*–*8*). The Centers for Disease Control and Prevention (CDC) guidelines now recommend that patients with clinical evidence of systemic anthrax disease receive an antitoxin agent in combination with antimicrobial agents (*9*).

Anthrax immune globulin intravenous (AIG-IV; current trade name Anthrasil, manufactured by Emergent BioSolutions Inc., Rockville, MD, USA) is one of the few antitoxin agents approved by the Food and Drug Administration (FDA) and included in the Strategic National Stockpile (*10*). It is a polyclonal human antibody prepared from the serum of persons previously vaccinated with anthrax vaccine adsorbed (BioThrax; Emergent BioSolutions, Gaithersburg, MD, USA). Because of the infrequency of invasive *B. anthracis* infection, AIG-IV approval was based on its efficacy in anthrax animal models in combination with safety data from healthy humans (*11*,*12*). Therefore, although AIG-IV has been the only antitoxin therapy administered clinically since the 2001 US anthrax outbreak, its actual efficacy in humans is unknown. Reviewing clinical experiences where AIG-IV has been administered for anthrax is important to inform future use of this agent and of antitoxin agents in general.

Although AIG-IV use has been reported in 3 isolated anthrax cases (*13*–*16*), the largest clinical experience with it came during an outbreak of *B. anthracis* soft tissue infection in injection drug users in the United Kingdom during 2009–2010. These cases were secondary to heroin injections contaminated with the same *B. anthracis* strain (*17*–*24*). Of the 52 confirmed cases in this outbreak, 47 occurred in Scotland, and 15 of these persons received AIG-IV through the coordinated efforts of CDC, Health Protection Scotland (HPS), and the Scottish National Anthrax Outbreak Control Team. Although the epidemiology of this outbreak and the clinical characteristics of a subgroup of 27 patients has been reported, a review of experience with AIG-IV itself and its effects on recovery has not (*18*,*25*). Here we examine that experience in 15 recipients and 28 nonrecipients of the agent.

## Methods

### Approval

This study used de-identified data collected during routine hospital care of patients. The Office of Human Subjects Research from the Clinical Center at the National Institutes of Health (Bethesda, MD, USA) determined the study to be exempt from institutional review board review.

### AIG-IV Availability, Distribution, and Administration

Representatives of CDC and the Scottish National Anthrax Outbreak Control Team directly involved with the 2009–2010 UK anthrax outbreak provided information about how AIG-IV was distributed and administered. Data from a previously published survey of physicians caring for patients during the outbreak were also reviewed (*18*).

### Clinical Characteristics and Outcomes Comparing AIG-IV Recipients and Nonrecipients

Data regarding the clinical characteristics and outcomes of patients came from 2 sources. One was clinical data CDC obtained under its AIG-IV emergency investigational new drug application (E-IND). The other was from a previous survey of physicians caring for patients during the outbreak that had sought information about the disease characteristics, care, and outcome of patients (*18*). This previous survey did not compare AIG-IV recipients and nonrecipients.

### Lethal Factor Level Determinations

Lethal factor (LF) toxemia was quantified at CDC’s Clinical Chemistry Branch, Division of Laboratory Sciences (Atlanta, GA, USA), by using a validated mass spectrometry method that reports specific LF endoproteinase activity in nanograms per milliliter of serum. The LF mass spectrometry assay had precision of 8%–14%, accuracy of 92%–98%, and 100% diagnostic sensitivity and specificity (*26*).

### Data Analysis

We analyzed parameters for which >50% of patients had data reported for that parameter. The sequential organ failure assessment (SOFA) scores analyzed were those recorded by physicians caring for patients (*27*). Categorical data were analyzed with χ^2^ if not sparse or with Fisher exact test if otherwise. Normally distributed continuous data were analyzed by calculating the mean ± SE and compared between groups by using 2-sample *t* tests. Otherwise, data were summarized with medians (interquartile range [IQR]). Times from exposure to symptom onset; from symptom onset to hospital admission; and from hospital admission to anthrax diagnosis, surgery, AIG-IV administration, and intensive care unit (ICU) or hospital discharge were compared with Wilcoxon rank sum tests, and physical and laboratory findings not normally distributed were log-transformed and then compared by using 2-sample *t* tests. To assess the trend of LF over time, we used a linear regression model with a common slope and different intercepts for survivors and nonsurvivors. LF levels were log_10_-transformed, and a random subject effect was used to account for repeated measures. We considered 2-sided p values <0.05 to be significant without adjusting for multiple comparisons. All analysis were done by using SAS version 9.3 (SAS Institute, Cary, NC, USA).

## Results

### Outbreak

During the *B. anthracis* outbreak in the United Kingdom during December 9, 2009–July 12, 2010, a total of 47 confirmed cases were reported in Scotland from 14 hospitals. A case was defined as confirmed on the basis of a positive bacterial culture or PCR result from blood or tissue or on paired serology samples showing increasing antibody protective antigen or LF titers (*25*).

### AIG-IV Availability, Distribution, and Administration

During the outbreak, 15 patients received AIG-IV under the CDCs E-IND, and all treatments were from the same AIG-IV batch (Cangene Corp., Winnipeg, MB, Canada). CDC’s Division of Strategic National Stockpile provided the first set of AIG-IV doses for use on December 18, 2009, and a limited number of AIG-IV doses were available throughout the remainder of the outbreak. HPS distributed AIG-IV during the outbreak. Clinicians identified patients for AIG-IV treatment in accordance with criteria outlined in the E-IND and published by HPS (*28*). To be eligible to receive AIG-IV, patients had to have laboratory confirmation of *B. anthracis* infection based on positive cultures or PCR results; visualization of gram-positive bacilli from blood, tissue, or a normally sterile site; or other confirmed evidence of anthrax (e.g., positive paired serologic results as noted earlier) consistent with *B. anthracis* infection (Table 1). Having met these criteria, however, whether a patient was administered AIG-IV was at the discretion of the treating physicians and their impression that a patient would or would not benefit from this treatment.

**Table 1:** Clinical criteria for administering anthrax immune globulin intravenous during an outbreak of anthrax in injection drug users, Scotland, UK, 2009–2010

Criteria
1. Systemic illness in a heroin user with >1 of the following: a. Severe cellulitis, especially accompanied by substantial soft tissue edema b. Sudden onset of sepsis with no other obvious source c. Meningitis, which might also be characterized by subarachnoid hemorrhage d. Respiratory symptoms (suspect inhalational anthrax) e. Gastrointestinal symptoms (suspect gastrointestinal anthrax);
OR
2. Features clinically compatible with cutaneous, inhalation, or gastrointestinal illness with systemic effects (including malaise, myalgias, or fever).
In addition to 1 or 2:
Laboratory confirmation by isolation or visualization of a gram-positive bacillus consistent with *Bacillus* *anthracis* from blood, tissue, or a normally sterile site or other laboratory-confirmed evidence of anthrax infection after discussion with a local microbiologist or the Special Pathogens Reference Laboratory, Health Protection Agency, Porton Down, UK;
AND
An epidemiologic link to a documented anthrax exposure (such as being a heroin injecting drug user).

Under the E-IND, patients receiving AIG-IV were assessed, and baseline status was recorded before AIG-IV administration. All patients received a single similar dose (420 U) of AIG-IV. Patients were then monitored during and after the infusion until discharge. Monitoring during infusion frequently occurred in the ICU.

### Initial Clinical Characteristics of AIG-IV Recipients and Nonrecipients

**Figure 1:**
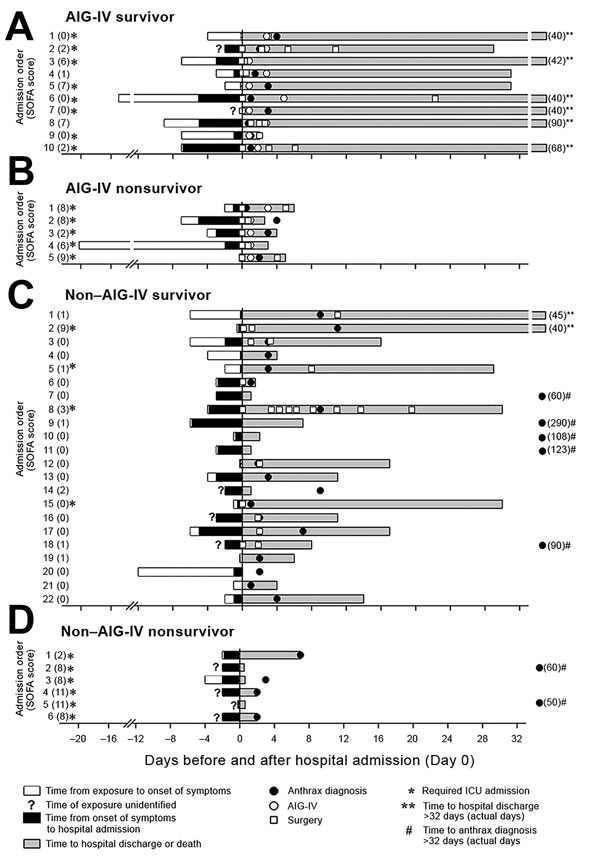
Key events during the illness courses of 15 patients who received AIG-IV (10 survivors, 5 nonsurvivors) and 28 patients who did not receive AIG-IV (22 survivors, 6 nonsurvivors) from the time of their suspected exposure to contaminated heroin to the time of discharge from hospital or to death, Scotland, UK, 2009–2010. A) AIG-IV recipient who survived. B) AIG-IV recipient who died. C) AIG-IV nonrecipient who survived. D) AIG-IV nonrecipient who died. AIG-IV, anthrax immune globulin intravenous; ICU, intensive care unit; SOFA, sequential organ failure.

For 43 patients, data were available for review and analysis, including for all 15 AIG-IV recipients. Times from contaminated heroin exposure to symptom onset and from symptom onset to hospitalization did not differ significantly between AIG-IV recipients and nonrecipients (Figure 1; Table 2). Age, sex, smoking status, excessive alcohol use, hepatitis C status, and use of different routes or limbs for drug injection did not differ significantly (Table 2). The proportion of patients who had only localized skin or limb complaints or only generalized complaints or a combination of localized and generalized complaints did not differ significantly between AIG-IV recipients and nonrecipients (Table 2). Among AIG-IV recipients, neither the time from exposure to AIG-IV treatment between survivors and nonsurvivors (median [IQR] 8 [6.5–11.0] days vs. 5.5 [3.0–7.0] days; p = 0.12] nor the time from symptom onset to treatment (4 [1–8] days vs. 4 [3–5] days; p = 0.59] differed significantly.

**Table 2:** Medical history of AIG-IV recipients and nonrecipients, Scotland, UK, 2009–2010*

Variable	AIG-IV nonrecipient	AIG-IV recipient	p value
Presentation and clinical history			
Days from exposure to symptom onset, median (IQR)	1 (0.0–4.0), n = 18	2 (1.0–4.5), n = 12	0.19
Days from symptom onset to hospitalization, median (IQR)	2 (1.0–3.0), n = 25	2.0 (1.0–5.0), n = 14	0.55
Age, y, mean ± SE	34.2 ± 1.5, n = 28	37.5 ± 1.6, n = 15	0.18
Male sex	60.7 (17/28)	73.3 (11/15)	0.41
Smoker	94.4 (17/18)	81.8 (9/11)	0.54
Alcohol user	33.3 (5/15)	58.3 (7/12)	0.19
Hepatitis C infection	60 (9/15)	77.8 (7/9)	0.66
Drug injection route and site			
Intravenous	50 (14/18)	40 (6/15)	0.53
Intramuscular	21.4 (6/28)	46.7 (7/15)	0.16
Arm	39.3 (11/28)	26.7 (4/15)	0.41
Groin	25 (7/28)	26.7 (4/15)	1.00
Buttock	7.1 (2/28)	26.7 (4/15)	0.16
Leg	10.7(3/28)	6.7 (1/15)	1.00
Presenting complaints			
Local†	67.9 (19/28)	53.3 (8/15)	0.54
General‡	17.9 (5/28)	20.0 (3/15)	
Both%	14.3 (4/28)	26.7 (4/15)	

*Values are % patients (no. patients with the finding/no. patients for whom data were available) except as indicated. n values indicate number of patients for whom data were available. AIG-IV, anthrax immune globulin intravenous; IQR, interquartile range. †Skin lesion, limb swelling, or limb pain. ‡Fever, diaphoresis, confusion, seizures, lethargy, or malaise.

On initial physical examination, AIG-IV recipients had lower body temperature than nonrecipients (mean ± SE 36.3°C ± 0.4 vs. 37.3°C ± 0.3; p = 0.05), but heart and respiratory rates and blood pressure, and Glasgow coma scores (GCS) did not differ significantly (Table 3). Although a smaller proportion of recipients had limb edema (7 [47%] of 15 vs. 21 [84%] of 25; p = 0.03] and limb pain (7 [47%] of 15 vs. 17 [89%] of 19; p = 0.01), neither the presence nor type of skin finding differed significantly between the groups (Table 3). Although a greater proportion of nonrecipients had confusion (6 [35%] of 17 vs. 0 of 13; p = 0.02), other nonskin and nonlimb findings did not differ significantly between groups.

**Table 3:** Initial physical findings of recipients and nonrecipients of AIG-IV, Scotland, UK, 2009–2010*

Physical finding	AIG-IV nonrecipient	AIG-IV recipient	p value
Vital signs [reference value]†			
Temperature, °C, mean ± SE [36.1–37.2°C]	**37.3 ± 0.3, n = 28**	**36.3 ± 0.4, n = 15**	**0.05**
Systolic BP, mmHg, mean ± SE [90–140 mm Hg]	113.6 ± 3.8, n = 28	117.2 ± 5.0, n = 15	0.57
Diastolic BP, mmHg, mean ± SE [60–90 mm Hg]	65.9 ± 3.0, n = 28	68.6 ± 4.3, n = 15	0.59
Mean BP, mmHg, median (IQR) [70–100 mm Hg]	83.5 (74.3–90.7), n = 28	88.0 (67.7–93.3), n = 15	0.57
Heart rate, beats/min, mean ± SE [60–100 beats/min]	104.0 ± 4.7, n = 28	102.4 ± 5.3, n = 15	0.83
Respiratory rate, breaths/min, mean ± SE [12–20 breaths/min]	18.8 ± 1.4, n = 28	19.9 ± 1.7, n = 13	0.64
Glasgow coma score, median (IQR) [15]	15 (15–15), n = 28	15 (15–15), n = 15	0.56
Skin and limbs			
Skin lesion	85.2 (23/27)	73.3 (11/15)	0.43
Ulcer	35.3 (6/17)	10 (1/10)	0.20
Exude	27.8 (5/18)	18.2 (2/11)	0.68
Limb mottling	26.7 (4/15)	60 (6/10)	0.12
Eschar	17.7 (3/17)	18.2 (2/11)	1.00
Local pain	100 (19/19)	85.7 (12/14)	0.17
Localized edema	95.8 (23/24)	93.3 (14/15)	1.00
Local erythema	87.0 (20/23)	92.3 (12/13)	1.00
Limb pain	**89.5 (17/19)**	**46.7 (7/15)**	**0.01**
Limb edema	**84 (21/25)**	**46.7 (7/15)**	**0.03**
Findings other than skin and limb			
Fever	60.9 (14/23)	66.7 (8/12)	1.00
Diaphoresis	63.6 (7/11)	40 (4/10)	0.39
Lethargy	64.3 (9/14)	41.7 (5/12)	0.25
Nausea	29.4 (5/17)	30 (3/10)	1.00
Abdomen pain	6.7 (1/15)	20 (2/10)	0.54
Confusion	**35.3 (6/17)**	**0 (0/13)**	**0.02**

*Values are % patients (no. patients with the finding/no. patients for whom data were available) except as indicated. n values indicate no. patients for whom data were available. Bold indicates statistical difference between AIG-IV recipients and nonrecipients. AIG-IV, anthrax immune globulin intravenous; BP. blood pressure; IQR, interquartile range.

We compared results of initial laboratory results of AIG-IV recipients and nonrecipients. Recipients had higher total levels than nonrecipients for leukocytes (median [IQR] 18.9 [9.5–23.2] vs. 10.9 [8.6–14.1] cells × 10^3^ cells/μL; p = 0.02); neutrophils (15.4 [7.4–19.5] vs. 7.6 [5.2–10.0] × 10^3^ cells/μL; p = 0.008); blood urea nitrogen (8.6 [7.1–13.9] vs. 4.3 [3.7–6.0] mmol/L; p = 0.01); creatinine (102.0 [84.0–189.0] vs. 75.5 [64.0–89.5] mmol/L; p = 0.04); and bilirubin (13.5 [9.0–17.0] vs. 8.0 [5.0–11.0] μmol/L; p = 0.02) but lower levels for bicarbonate (mean ± SE 20.7 ± 0.9 vs. 24.4 ± 1.2 mmol/L; p = 0.02); alkaline phosphatase (85 [56–97] vs. 100 [74–189] U/L; p = 0.04); total protein (46 [41–62] vs. 67 [65–75] g/L; p = 0.001); and albumin 27.6 ± 2.6 vs. 38.3 ± 1.1 g/L; p<0.0001) levels (Table 4). Other laboratory parameters did not differ significantly between the 2 groups (Table 4).

**Table 4:** Initial laboratory findings of AIG-IV recipients and nonrecipients, Scotland, UK, 2009–2010*

Laboratory test [reference value]	Non-AIG-IV	AIG-IV	p value
Complete blood counts and differentials			
Leukocytes, × 10^9^ cells/L, median (IQR) [4–11 × 10^9^/L]	**10.9 (8.6–14.1), n = 27**	**18.9 (9.5–23.2), n = 15**	**0.02**
Neutrophils, × 10^9^ cells/L, median (IQR) [2–7 × 10^9^/L]	**7.6 (5.2–10.0), n = 26**	**15.4 (7.4–19.5), n = 15**	**0.008**
Lymphocytes, × 10^9^ cells/L, median (IQR) [1–3 × 10^9^/L]	1.8 (1.3–2.5), n = 25	2.0 (1.3–2.7), n = 15	0.78
Hemoglobin, g/dL, mean ± SE [12–18 g/dL]	14.0 ± 0.8, n = 27	14.8 ± 1.5, n = 15	0.61
Hematocrit, %, mean ± SE [35%–50%]	41 ± 2, n = 23	42 ± 4, n = 13	0.86
Platelets, × 10^9^/L, mean ± SE [150–450 × 10^9^/L]	214 ± 19, n = 24	181 ± 24, n = 15	0.29
Coagulation parameters and C-reactive protein			
Prothrombin time(s), median (IQR) [11–13.5 s]	11.0 (10.0–14.0), n = 15	12.8 (12.0–15.0), n = 14	0.94
Partial thromboplastin time(s), median (IQR) [25–35 s]	26.7 (24.0–36.0), n = 10	33.8 (30.0–39.0), n = 14	0.63
International normalized ratio, median (IQR) [0.8–1.1]	1.1 (1.0–1.3), n = 11	1.1 (1.0–1.3), n = 11	0.36
C-reactive protein, nmol/L, median (IQR) [<95 nmol/L]	21 (8–49), n = 25	32 (17–52), n = 14	0.24
Serum electrolytes and glucose			
Sodium, mmol/L, median (IQR) [135–145 mmol/L]	137 (132–139), n = 27	135 (131–136), n = 15	0.11
Chloride mmol/L, median (IQR) [96–108 mmol/L]	100 (96–101) , n = 13	102 (101–103), n = 14	0.76
Potassium, mmol/L, mean ± SE [3.5–5.3 mmol/L]	4.26 ± 0.13, n = 28	4.35 ± 0.22, n = 13	0.69
Calcium, mmol/L, median (IQR) [2.25–2.5 mmol/L]	2.3 (2.0–2.3), n = 28	2.1 (2.0–2.3), n = 12	0.97
HCO3^-^ , mmol/L, mean ± SE [22–28 mmol/L]	**24.4 ± 1.2, n = 11**	**20.7 ± 0.9, n = 11**	**0.02**
Glucose, mmol/L, median (IQR) [3.6–6.0 mmol/L]	6.5 (5.6–8.1), n = 16	7.8 (5.3–8.9), n = 10	0.69
Renal and liver functions			
Blood urea nitrogen, mmol/L, median (IQR) [2.5–7.8 mmol/L]	**4.3 (3.7–6.0), n = 28**	**8.6 (7.1–13.9), n = 15**	**0.01**
Creatinine, mmol/L, median (IQR) [40–130 μmol/L]	**75.5 (64.0–89.5), n = 28**	**102.0 (84.0–189.0), n = 15**	**0.04**
Bilirubin, μmol/L, median (IQR) [5–17 μmol/L]	**8.0 (5.0–11.0), n = 25**	**13.5 (9.0–17.0), n = 14**	**0.02**
Alanine aminotransferase, U/L, median (IQR) [<50 U/L]	18.5 (14–36.5), n = 16	28.0 (11.0–40.0), n = 14	0.69
Alkaline phosphatase, U/L, median (IQR) [30–130 U/L]	**100 (74–189), n = 15**	**85 (56–97), n = 11**	**0.04**
Total protein, g/L, median (IQR) [60–80 g/L]	**67 (65–75), n = 12**	**46 (41–62), n = 13**	**0.001**
Albumin, g/L, mean ± SE [35–55 g/L]	**38.3 ± 1.1, n = 25**	**27.6 ± 2.6, n = 14**	**<0.0001**

*n values indicate no. patients for whom data were available. Bold indicates significant differences between AIG-IV recipients and nonrecipients. AIG-IV, anthrax immune globulin intravenous; IQR, interquartile range.

On microbiological examination, a higher proportion of AIG-IV recipients than nonrecipients had positive blood cultures (10 [71%] of 14 vs. 8 [32%] of 25; p = 0.02) and positive blood PCR results (8 [80%] of 10 vs. 5 [29%] of 17; p = 0.02) for *B. anthracis* (Table 5). Other microbiological data did not differ significantly between the 2 groups (Table 5). The time to anthrax diagnosis was shorter for AIG-IV recipients than for nonrecipients (median [IQR] 2.0 [1.0–3.0] vs. 3.5 [2.0–30.5]; p = 0.006] (Figure 1; Table 5).

**Table 5:** Microbiology data and the time to confirmatory anthrax diagnosis for recipients and nonrecipients of AIG-IV, Scotland, UK, 2009–2010*

Laboratory test	AIG-IV nonrecipient	AIG-IV recipient	p value
Blood culture	**32 (8/25)**	**71.4 (10/14)**	**0.02**
Wound culture	46.2 (6/13)	33.3 (3/9)	0.67
Tissue culture	54.6 (6/11)	70 (7/10)	0.66
Blood PCR	**29.4 (5/17)**	**80 (8/10)**	**0.02**
Blood protective antigen antibody	81.3 (13/16)	66.7 (4/6)	0.59
Blood lethal factor antibody	62.5 (10/16)	57.1 (4/7)	1.00
Days to diagnosis, median (IQR)	**3.5 (2.0–30.5), n = 28**	**2.0 (1.0–3.0), n = 13**	**0.006**

*Values are % patients (no. patients with the finding/no. patients for whom data were available) except as indicated. n values indicate no. patients for whom data were available. Bold indicates significant differences between AIG-IV recipients and nonrecipients. AIG-IV, anthrax immune globulin intravenous; IQR, interquartile range.

### Treatments of AIG-IV Recipients and Nonrecipients

The median time to AIG-IV treatment in recipients was 1 day (IQR 1–3 days) (Figure 1; Table 6). No adverse events were documented during AIG-IV administration. A greater proportion of AIG-IV recipients than nonrecipients had surgery either on the day of (11 [73%] of 15 vs. 5 [18%] of 28; p = 0.0003) or at any time during [14 [93%] of 15 vs. 11 [39%] of 28; p = 0.0006] hospital admission (Figure 1; Table 6). AIG-IV recipients received more types of antimicrobial drugs than did nonrecipients (mean ± SE 5.3 ± 0.2 vs. 3.0 ± 0.2; p < 0.0001). Administration of mechanical ventilation or vasopressors did not differ between the groups (p>0.36; data not shown).

**Table 6:** Treatment after hospital admission and durations of ICU and hospital stay in survivors and nonsurvivors who did and did not receive AIG-IV, Scotland, UK, 2009–2010*

Treatment characteristic	AIG-IV nonrecipient	AIG-IV recipient	p value
Treatments after hospital admission			
Days to AIG-IV receipt, median (IQR)	NA	1 (1–3), n = 15	NA
ICU care	**35.7 (10/28)**	**86.7 (13/15)**	**0.001**
Receipt of antimicrobial drugs	100 (28/28)	100 (15/15)	1.00
No. antimicrobial drugs/patient during hospital stay, mean ± SE‡	**3.0 ± 0.2, n = 28**	**5.3 ± 0.2, n = 15**	**<0.0001**
Surgery on day of admission	**17.9 (5/28)**	**73.3 (11/15)**	**0.0003**
Surgery during hospital stay	**39.3 (11/28)**	**93.3 (14/15)**	**0.0006**
Days to surgery, median (IQR)	1 (0–2), n = 11	0 (0–0.33), n = 14	0.24
Vasopressors	13.3 (2/15)	33.3 (4/12)	0.36
Mechanical ventilation	33.3 (5/15)	50 (7/14)	0.36
Duration of ICU and hospital stay, median (IQR)			
Survivors’ ICU stay, d	**0, n = 22**	**4.5 (0.9–19.0), n = 10**	**0.0008**
Nonsurvivors’ time to death, d	**1.3 (0.6–2.0), n = 6**	**4.0 (3.0–5.0), n = 5**	**0.07**
Survivors’ hospital stay, d	**9.5 (2.0–17), n = 22**	**38 (31–42), n = 10**	**0.001**

*Values are % patients (no. patients with the finding/no. patients for whom data were available) except as indicated. n values indicate no. patients for whom data were available. Bold indicates significant differences between AIG-IV recipients and nonrecipients. AIG-IV, anthrax immune globulin intravenous; ICU, intensive care unit; IQR, interquartile range; NA, not applicable.

### Outcomes

**Figure 2:**
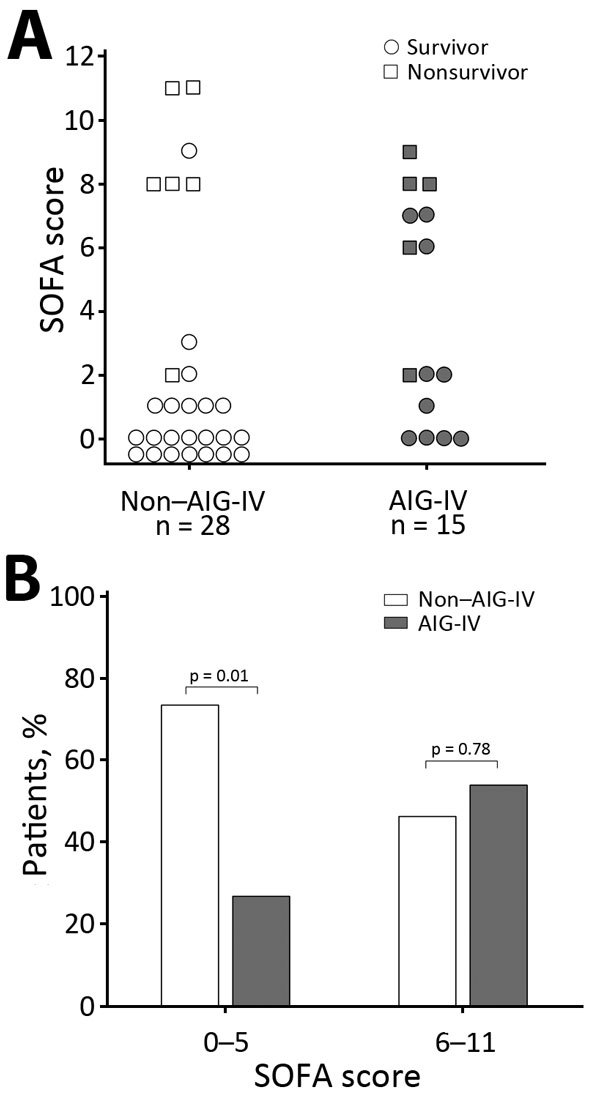
SOFA scores of injection drug users who did and did not receive AIG-IV, Scotland, UK, 2009–2010. A) Individual SOFA scores for patients who did or did not receive AIG-IV and did or did not survive. B) Proportion of patients who did or did not receive AIG-IV for those who had admission SOFA scores of 0–5 and a low risk for death and for those with SOFA scores of 6–11 and a higher risk for death. AIG-IV, anthrax immune globulin intravenous; SOFA, sequential organ failure.

Five (33%) of 15 AIG-IV recipients and 6 (21%) of 28 nonrecipients died, and these death rates did not differ significantly (p = 0.47) (Figure 1). However, in patients overall, risk for death at admission as reflected by the SOFA score was greater for AIG-IV recipients than for nonrecipients, although this finding did not reach statistical significance (median [IQR] 2 [0–7] vs. 0.5 [0–2.5]; p = 0.14). However, SOFA scores were not distributed equally between recipients and nonrecipients (Figure 2). Of the 30 patients with a SOFA score of 0–5 (indicating a low risk for death), only 8 (27%) received AIG-IV (p = 0.01, against the null hypothesis that 50% of these patients received AIG-IV). On the other hand, of the 13 patients with a SOFA score of 6–11 (indicating a higher risk for death), 7 (54%) received AIG-IV (p = 0.78, against the null hypothesis that 50% of these patients received AIG-IV). Death rates did not differ between AIG-IV recipients and nonrecipients either for patients with SOFA scores of 0–5 (1 [13%] nonsurvivor of 8 recipients vs. 1 [5%] of 22 nonrecipients; p = 0.47) or for patients with SOFA scores of 6–11 (4 [57%] of 7 recipients vs. 5 (83%) of 6 nonrecipients, p = 0.56]. For patients with SOFA scores of 6–11, the median score was higher in nonrecipients than recipients (8.5 [8–11] vs. 7 [6–8]; p = 0.03).

For survivors, duration of ICU and hospital stays were longer for recipients than for nonrecipients (median [IQR] for ICU stay, 4.5 [0.9–19.0] vs. 0 [0–0]; p = 0.0008; for hospital stay, 38.0 [31.0–42.0] vs. 9.5 [2.0–17.0]; p = 0.001) (Figure 1; Table 6). For nonsurvivors, the time to death was longer for recipients than for nonrecipients in a pattern approaching significance (4.0 [3.0–5.0] vs. 1.3 [0.6–2.0]; p = 0.07) (Figure 1; Table 6). Of the 6 nonsurvivors not receiving AIG-IV, 3 died within 24 h and 2 within 48 h after admission, times possibly too short for AIG-IV acquisition and treatment. Furthermore, nonsurvivors receiving AIG-IV had significantly higher GCS (better neurologic function) than nonrecipients (15 [15–15] vs. 9 [6–14]; p = 0.04). Consistent with this finding, all 4 head computed tomograms reported from patients in the outbreak were in nonrecipient nonsurvivors, and all showed evidence of subarachnoid hemorrhage (3 patients) or high attenuation material caused by subarachnoid hemorrhage or purulence (1 patient). Two of these nonrecipients died within 15 h after admission and 2 by 48 h. In addition, although all nonsurvivors receiving AIG-IV had at least 1 surgery, no nonsurvivor not receiving AIG-IV had surgery (Figure 1).

### Four Patients from the Outbreak for Whom Data Were Unavailable

None of the 4 patients for whom data were unavailable received AIG-IV. Of these, 2 survived and 2 did not (*25*). Therefore, across all 47 patients in Scotland, the proportion of patients dying did not differ significantly between AIG-IV recipients and nonrecipients (5 [33%] of 15 vs. 8 [25%] of 32; p = 0.73).

### LF Levels in AIG-IV Recipients

**Figure 3:**
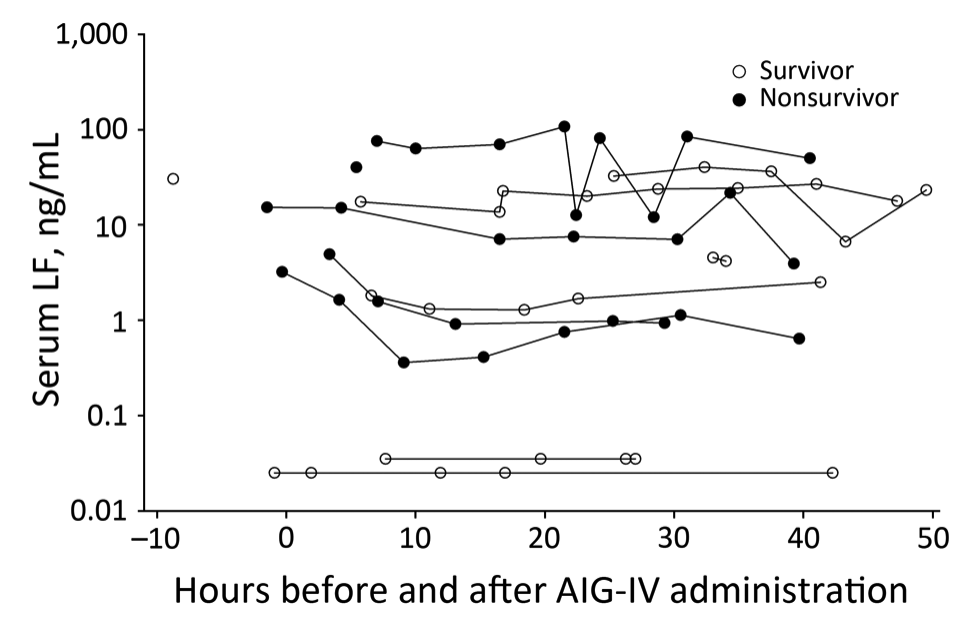
Individual LF levels (nanograms per milliliter) in 12 anthrax patients receiving AIG-IV from 10 h before until 50 h after treatment administration, Scotland, UK, 2009–2010. AIG-IV, anthrax immune hlobulin intravenous; LF, lethal factor.

LF levels were available for 5 nonsurvivors and 7 survivors receiving AIG-IV and from no nonrecipients. These levels were examined over the period they were available for both nonsurvivors and survivors (10 h before and up to 50 h after AIG-IV administration). Before AIG-IV treatment, LF levels trended to be higher but did not differ significantly between nonsurvivors and survivors (p = 0.42) (Figure 3). Two survivors had LF levels <0.1 ng/mL, noticeably lower than levels of other patients. After AIG-IV treatment, levels trended slightly lower and with a common slope approaching significance (p = 0.08).

## Discussion

The experience with AIG-IV during the 2009–2010 anthrax outbreak in injection drug users in Scotland is, to our knowledge, the largest single experience with this agent. Despite AIG-IV efficacy in animal models (*11*,*12*), death rates did not differ significantly between 15 patients who did and the 28 who did not receive therapy. However, several criteria indicated that AIG-IV recipients were sicker than nonrecipients, and this difference confounds an assessment of the efficacy of AIG-IV.

Although SOFA scores seemed to suggest that AIG-IV recipients were at higher risk than nonrecipients for death, this trend did not reach significance (p = 0.14). However several laboratory findings differed significantly between the 2 groups and were consistent with more severe disease in AIG-IV recipients: lower temperature, serum bicarbonate, total protein, and albumin and higher leukocytes, neutrophils, blood urea nitrogen, creatinine, and bilirubin. Patients with a low risk for death (SOFA score <5) were less likely to receive AIG-IV (8 of 30; p = 0.01). In contrast, the proportions of recipients and nonrecipients with a higher risk for death (SOFA scores >6) did not differ. The number of patients with higher SOFA scores was too small to assess AIG-IV effects in this subgroup. Consistent with the possibility that AIG-IV recipients were sicker than nonrecipients, among survivors, ICU and hospital stay were significantly longer for recipients, suggesting longer recovery from more severe disease. More AIG-IV recipients than nonrecipients had surgery to manage their disease, and a greater proportion of AIG-IV recipients had blood cultures positive for *B. anthracis*, suggesting a higher bacterial load.

For at least 3 reasons, AIG-IV treatment might have been directed to more severely ill patients. First, the sensitivity of various diagnostic laboratory criteria for documenting anthrax can vary on the basis of the severity of infection. Positive blood cultures, suggestive of significant bacterial load, often were available early during the patient’s course (i.e., within <1 day), resulting in timely consideration of AIG-IV. In contrast, confirmation based solely on paired serum samples, reflecting less severe infection, required far longer for results to be available (often weeks), thus mitigating against AIG-IV use. Second, criteria for AIG-IV stipulated that patients have evidence of systemic and therefore more severe illness. Third, outbreak treatment teams reported in personal communications to authors of this article (L.N. and M.B.) that AIG-IV was considered a limited resource and that treatment was directed to patients with evidence of more severe infection but with a likelihood of survival.

For at least 2 possible reasons, some patients with high SOFA scores did not receive AIG-IV. First, disease might have progressed too quickly in some nonsurvivors for AIG-IV to be made available; 3 nonrecipients died within 24 h after seeking care and 2 within 48 h. Second, among nonsurvivors, nonrecipients had significantly poorer neurologic status at admission, as assessed by GCS scores, than did AIG-IV recipients, and none underwent surgery, suggesting that care might have been limited in these patients. In fact, 4 of these patients had evidence of subarachnoid hemorrhage soon after seeking care. In personal communications, caregivers reported withholding AIG-IV in patients with severe neurologic deficits.

Thus, on the basis of experience during this outbreak in injection drug users, whether AIG-IV provides benefit for anthrax-related soft tissue infection is unclear. This form of infection has only recently been identified and has received little preclinical study. Debridement was effective in a mouse model of subcutaneous anthrax, but that study did not investigate the efficacy of antitoxin therapies (*29*). The preclinical models on which FDA based its approval of AIG-IV all used aerosolized bacterial challenge to simulate inhalational anthrax (*11*,*12*). In these studies, AIG-IV added to the protective effects of antimicrobial drugs when both treatments were administered after the onset of lethality, in trends that approached significance (*11*). The prior clinical experience with AIG-IV also has been restricted to inhalational disease (3 cases) or gastrointestinal disease (1 case) contracted by inhalation or ingestion of spores (*13*–*16*). Although 3 of these 4 patients survived, an overall survival rate higher than previously reported with these forms of disease, to what extent this might have been related to AIG-IV treatment or other factors is unknown. Studies examining the effects of AIG-IV or other antitoxin agents when combined with antimicrobial drugs and debridement in animal models are necessary to better define the optimal therapeutic approach for this newly identified form of anthrax. Prompt and aggressive antimicrobial therapy and surgical debridement if necrotic tissue requires it, remain the mainstays of management for soft tissue infection when anthrax is suspected. However, on the basis of animal efficacy studies with other forms of anthrax, as well as human safety studies, AIG-IV or other approved antitoxin agents still might be considered as adjunctive therapy when clinical suspicion is high for systemic anthrax.

LF levels trended higher in nonsurvivors than survivors before AIG-IV administration, but this difference was not significant. Although there was a small but close to significant reduction in LF levels after AIG-IV administration, without data from nonrecipients, determining whether this decline reflects the effect of antibody treatment or the course of the infection itself is not possible. LF levels could have been influenced by previous antimicrobial use. Although current LF detection is based on serum and plasma levels, the relationship between these and tissue levels is currently under investigation. Tissue levels ultimately might be more instructive than serum and plasma levels for disease treatment.

No adverse events related to AIG-IV administration were reported to CDC during this *B. anthracis* outbreak. The number of recipients was relatively small, however, and given these patients’ severity of disease, identifying adverse effects of treatment without an equivalent control group would be difficult.

This study has limitations. First, data were obtained retrospectively and not completely for all patients. However, parameters were analyzed and presented only if available from >50% of patients. Furthermore, the SOFA score on which stratification of AIG-IV treatment and survival data was based, and which is a well-regarded gauge of disease severity and lethality risk (*27*), was available for all 43 patients analyzed. Second, data were not available regarding decisions about whether individual patients should or should not receive AIG-IV treatment. Third, the overall number of patients available for analysis was small. However, our analysis addressed the single largest experience with an antitoxin agent for treating anthrax since the introduction of antimicrobial drugs during the 1940s and the routine use of modern ICU support in the early1960s (*30*).

In conclusion, guidelines now recommend treatment with agents inhibiting the effects of lethal and edema toxins for patients with a high likelihood of having systemic anthrax infection. AIG-IV is one of the few antitoxin agents that has received FDA approval and been included in the Strategic National Stockpile. Documenting the clinical experience with anthrax antitoxin agents is critical for further defining this therapeutic approach. Whether AIG-IV treatment is effective for systemic anthrax soft tissue infection related to drug injection cannot be answered with currently available data.
